# Interventions to de-implement unnecessary antibiotic prescribing for ear infections (DISAPEAR Trial): protocol for a cluster-randomized trial

**DOI:** 10.1186/s12879-023-08960-z

**Published:** 2024-01-24

**Authors:** Timothy C. Jenkins, Amy Keith, Amy B. Stein, Adam L. Hersh, Rashmi Narayan, Alice Eggleston, Deborah J. Rinehart, Payal K. Patel, Eve Walter, Ian G. Hargraves, Holly M. Frost, for the DISAPEAR Study Group, Leisha Andersen, Shaun Cosgrove, Aiden Gilbert, Hannah Jensen, Theresa Morin, Barbora Nelson, Allan M. Seibert, Valoree Stanfield, Park Willis

**Affiliations:** 1 Division of Infectious Diseases, Department of Medicine, Denver Health and Hospital Authority, Denver, CO USA; 2 Division of Infectious Diseases, Department of Medicine, University of Colorado School of Medicine, Aurora, CO USA; 3 Center for Health Systems Research, Denver Health and Hospital Authority, 601 Broadway Ave, Denver, CO USA; 4 Division of Infectious Diseases, Department of Pediatrics, University of Utah, Salt Lake City, UT USA; 5 AllianceChicago, Chicago, IL USA; 6 Division of General Internal Medicine, Department of Medicine, University of Colorado School of Medicine, Aurora, CO USA; 7 Division of Infectious Diseases and Clinical Epidemiology, Intermountain Health, Murray, UT USA; 8 Knowledge and Evaluation Research Unit, Mayo Clinic, Rochester, MN USA; 9 Department of Pediatrics, Denver Health and Hospital Authority, 601 Broadway Ave, Denver, CO USA; 10 Department of Pediatrics, University of Colorado School of Medicine, Aurora, CO USA

**Keywords:** Acute otitis media, Antibiotic stewardship, Implementation effectiveness, Watchful waiting

## Abstract

**Background:**

Watchful waiting management for acute otitis media (AOM), where an antibiotic is used only if the child’s symptoms worsen or do not improve over the subsequent 2–3 days, is an effective approach to reduce antibiotic exposure for children with AOM. However, studies to compare the effectiveness of interventions to promote watchful waiting are lacking. The objective of this study is to compare the effectiveness and implementation outcomes of two pragmatic, patient-centered interventions designed to facilitate use of watchful waiting in clinical practice.

**Methods:**

This will be a cluster-randomized trial utilizing a hybrid implementation-effectiveness design. Thirty-three primary care or urgent care clinics will be randomized to one of two interventions: a health systems-level intervention alone or a health systems-level intervention combined with use of a shared decision-making aid. The health systems-level intervention will include engagement of a clinician champion at each clinic, changes to electronic health record antibiotic orders to facilitate delayed antibiotic prescriptions as part of a watchful waiting strategy, quarterly feedback reports detailing clinicians’ use of watchful waiting individually and compared with peers, and virtual learning sessions for clinicians. The hybrid intervention will include the health systems-level intervention plus a shared decision-making aid designed to inform decision-making between parents and clinicians with best available evidence. The primary outcomes will be whether an antibiotic was ultimately taken by the child and parent satisfaction with their child’s care. We will explore the differences in implementation effectiveness by patient population served, clinic type, clinical setting, and organization. The fidelity, acceptability, and perceived appropriateness of the interventions among different clinician types, patient populations, and clinical settings will be compared. We will also conduct formative qualitative interviews and surveys with clinicians and administrators, focus groups and surveys of parents of patients with AOM, and engagement of two stakeholder advisory councils to further inform the interventions.

**Discussion:**

This study will compare the effectiveness of two pragmatic interventions to promote use of watchful waiting for children with AOM to reduce antibiotic exposure and increase parent satisfaction, thus informing national antibiotic stewardship policy development.

**Clinical trial registration:**

NCT06034080.

**Supplementary Information:**

The online version contains supplementary material available at 10.1186/s12879-023-08960-z.

## Background

Acute otitis media (AOM) is the most common reason children are prescribed antibiotics, affecting 5 million children and resulting in 10 million antibiotic prescriptions annually [1–3]. Though 84% of AOM episodes resolve without antibiotics [4, 5], antibiotics are prescribed to approximately 95% of children when AOM is diagnosed, [6, 7]. The use of unnecessary antibiotics contributes to the development of antibiotic-resistant organisms, making future infections more difficult to treat [8]. In fact, recent data indicates that antibiotic resistance is rapidly increasing among bacteria that commonly cause AOM [9]. Antibiotics also place children at risk for *C. difficile* infection [10], are associated with an increased risk for chronic diseases later in life [11–13], and reduce pediatric quality of life. More than a quarter of children receiving an antibiotic will experience an adverse drug event [14]. One way to reduce unnecessary antibiotic exposure is to use a watchful waiting approach for the treatment of AOM, where an antibiotic is started only if the child’s symptoms worsen or do not improve over the subsequent 2 to 3 days [15]. A watchful waiting approach may include: (1) observation, where symptoms are monitored and an antibiotic is prescribed at a later date if symptoms worsen or do not improve, or (2) a delayed antibiotic prescription, where an antibiotic is prescribed during the initial encounter, but parents are instructed to start the antibiotic only if symptoms worsen or do not improve. In previous clinical trials of children with AOM or other respiratory infections, watchful waiting was shown to reduce antibiotic use by over 62% and did not result in worsening of symptoms, a higher rate of complications, or reduced parent satisfaction [16–22]. Despite the results of these trials, fewer than 5% of children with AOM are managed with watchful waiting in clinical practice [7, 23–25].

The infrequent use of watchful waiting to manage AOM may be associated with barriers such as a lack of institutional guidance or clinical workflows to support this approach, concerns about the time and feasibility to communicate with families after the initial visit, and potential co-pays for additional visits. Furthermore, clinicians may prescribe an immediate antibiotic over watchful waiting because they believe parents expect an antibiotic; however, this is often an incorrect assumption [26, 27]. While most parents do believe an antibiotic is required to treat AOM, they also consistently report that pain management, not an antibiotic, is the most important aspect of their child’s AOM care [28]. As such, ensuring effective communication and discussion of treatment options between parents and clinicians during AOM management may improve care and reduce unnecessary antibiotic use.

One such method for promoting engagement between parents and clinicians is through shared decision-making [2]. Shared decision-making is an approach whereby parents and clinicians come to mutual agreement upon a treatment strategy when there is uncertainty about the most effective treatment approach or differing opinions about the optimal treatment. Shared decision-making is typically facilitated with the use of a conversation aid designed to inform the decision-making between parents and clinicians with best available evidence. A prior study of shared decision-making for AOM found that its use resulted in a 20% absolute reduction in antibiotic use and a 17% absolute increase in parent satisfaction [29]. While shared decision-making interventions may be effective tools to facilitate watchful waiting management, they have not been successfully scaled to improve care for children with AOM. Like shared decision-making interventions, health systems-level interventions have also led to reductions in prescribing for AOM; [23, 24, 30] however, studies comparing the effectiveness of health systems-level interventions and shared decision-making, or their use in combination, are lacking. Such studies are essential to develop appropriate guidance on the most effective approaches to minimize antibiotic exposure for children with AOM. The present study will compare the effectiveness and implementation outcomes of two pragmatic, patient-centered interventions to promote use of watchful waiting: a health systems-level intervention alone or a health systems-level intervention combined with shared decision-making. Both intervention approaches aim to reduce antibiotic exposure for children with AOM while increasing parent engagement and satisfaction with their child’s care.

## Methods

### Overview of study design

**Fig. 1:**
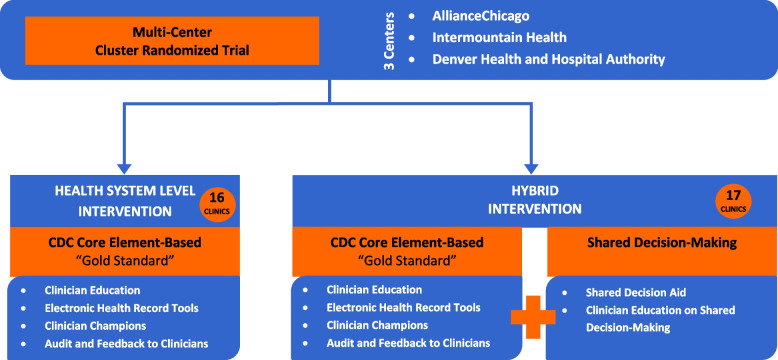
Cluster-randomized trial design

 This will be a pragmatic, cluster-randomized controlled trial that will use a hybrid implementation-effectiveness design [31]. The effectiveness and implementation outcomes of a “gold standard” [32] health systems-level intervention and a hybrid health systems-level plus shared decision-making intervention will be compared using the Practical Robust Implementation and Sustainability Model (PRISM) [33] and Reach Effectiveness Adoption Implementation Maintenance (RE-AIM) frameworks [34]. Participating clinics will be randomly assigned to one of the two interventions (Fig. 1) using covariate-constrained randomization. The primary outcomes of the study are 1) parent satisfaction with their child’s treatment for AOM, and 2) antibiotic courses *taken* by children with AOM by parent report. Secondary outcomes include 1) shared decision-making process and effectiveness [35, 36], 2) pediatric quality of life [14], 3) symptom severity and duration [22, 37, 38], 4) missed work, school, and daycare, 5) adverse drug events [14], 6) treatment failure [25, 39], 7) management strategy used (immediate antibiotic versus watchful waiting), and 8) whether or not an antibiotic prescription was filled at a pharmacy (Table 1).

**Table 1:** Primary and secondary outcome measures

Outcome	Metric^a^	Data Source	Time of Measurement(s) (Days)
Co-primary outcomes			
Antibiotic Taken	% filled and took antibiotic (yes/no)	Survey	10
Parent Satisfaction	% very or extremely satisfied- Likert scale (7- point) Validated	Survey	10
Secondary outcomes			
Shared decision-making (Process measure)	Summed Score- Knowledge and Decisional Conflict Assessment. Shared Decision-Making Questionnaire (SDM-Q-9)	Survey	0
Pediatric Quality of Life	PEDS-QL Score	Survey	10
Symptom Severity and Duration	Likert scale (7-point) Validated [22, 37]- all ages AOM-Severity of Symptom scale [38]- age 6–35 months	Survey	0, 10
1. Symptom severity (max and day 10)	Above	Survey	0, 10
2. Time to symptom improvement	Above	Survey	0, 10
3. Time to all symptom resolution	Above	Survey	0, 10
4. Time to ear pain resolution	Above	Survey	0, 10
5. Symptoms resolved at day 10	Above	Survey	10
Missed Work, School, Daycare	# Days missed school/daycare # Days parents missed work	Survey	10
Adverse Drug Events	% with adverse drug event(s)	Survey	10
Treatment Failure	% changed management (took antibiotic if initially watchful waiting or new antibiotic if initially immediate antibiotic)	Survey and EHR^b^	10
Management Strategy Used (Process Measure)	% immediate antibiotic	EHR	0
Antibiotic Filled (Process Measure)	% filled antibiotic (even if not taken)	Survey	10

^a^A comparable tool or measure may be substituted

^b^*EHR* Electronic health record

### Study setting and population

A total of 33 clinics from three geographically disparate health systems – AllianceChicago (AC) (a network of community health centers in Chicago, IL, USA), Denver Health and Hospital Authority (DHHA) (Denver, CO, USA), and Intermountain Health (IH) (Salt Lake City, UT, USA) – will be randomized. The participating clinics include over 200 pediatric, family medicine, and urgent care clinicians who serve more than 6,000 children with AOM annually (Table 2). The participating sites were selected to represent a diverse population that includes children of different races, ethnicities, language preferences, and socioeconomic statuses. Because most antibiotics for children in the United States are prescribed in non-academic settings, all participating clinics are community-based to increase the generalizability of study findings [21, 40].

**Table 2:** Aggregate data of clinic and patient characteristics for each participating site

**Clinic Characteristics**	**Alliance Chicago N (%)**	**Denver Health and Hospital Authority N (%)**	**Intermountain Healthcare N (%)**
Clinics to be randomized	10	9	14
Total eligible clinics	45	33	31
Family Medicine	0	7	0
Pediatric	0	23	0
Urgent Care	0	3	31
Hybrid (multi-specialty)	45	0	0
Clinical setting (participating)			
Urban	TBD	9 (100)	3 (10)
Suburban	TBD	0 (0)	24 (77)
Rural	TBD	0 (0)	4 (13)
Clinicians per clinic (mean eligible clinicians, range)	20.3 (3–51)	22.1(10–38)	29.3 (2–60)
Clinician Type			
Physician	TBD	123 (61.8)	205 (71.9)
Advanced Practice Provider	TBD	43(21.6)	80 (28.1)
Annual acute otitis media encounters per clinic (mean, range)	382 (121–1172)	228 (40–468)	346 (2–944)
**Patient Demographics**^a^	**N (%)** ***N* = 12,449**	**N (%)** ***N* = 7,524**	**N (%)** ***N* = 12,818**
Age (mean ± standard deviation, years)	4.3 (± 3.9)	3.4 (± 3.1)	5.2 (± 4.5)
Race			
American Indian/Alaskan Native	50 (0.4)	60 (0.8%)	91 (0.7%)
Asian	336 (2.7)	278 (3.7%)	152 (1.2%)
Black/African American	2042 (16.4)	1091 (14.5%)	163 (1.3%)
Native Hawaiian/Pacific Islander	212 (1.7)	45 (0.6%)	266 (2.1%)
White	7718 (62.0)	5477 (72.8%)	11,695 (91.2%)
Other/Unknown	2104 (16.9)	579 (7.7%)	451 (3.5%)
Ethnicity			
Hispanic/Latinx	7656 (61.5)	5116 (68.0%)	1,939 (15.1%)
Non-Hispanic/Latinx	4431 (35.6)	2408 (32.0%)	10,644 (83.1%)
Declined/Unknown			234 (1.8%)
Language Preference			
English	7108 (57.1)	4703 (62.5%)	12,556 (98.0%)
Spanish	5041 (40.5)	2332 (31.0%)	225 (1.8%)
Other	286 (2.3)	489 (6.5%)	37 (0.3)
Insurance			
Public (Medicaid, etc.)	9275 (74.5)	6629 (88.1%)	2406 (18.8%)
Commercial	3175 (25.5)	707 (9.4%)	9751 (76.1)
Uninsured	0 (0)	68 (0.9%)	661 (5.2%)
Unknown	0 (0)	120 (1.6%)	0 (0)

### Formative evaluation of context with PRISM

PRISM will be used to guide the formative implementation evaluation of the interventions. PRISM has demonstrated efficacy in identifying contextual factors to facilitate intervention adaptations to prevent program failure and increase program uptake across diverse settings. Assessment of external and internal factors using the PRISM domains of 1) Organizational Perspective, 2) Patient Perspective, 3) External Environment, 4) Implementation and Sustainability Infrastructure, 5) Organizational Characteristics, and 6) Patient Characteristics will be conducted using a mixed methods approach in the pre-implementation phase to make necessary program adaptions and guide implementation strategies (Table 3).

**Table 3:** Practical robust implementation and sustainability model (PRISM) components

PRISM domain	What are we assessing?	How will data be collected?	How will data be used?
Organizational perspective	• Preferred mechanisms to relay program changes and education to clinicians • Baseline antibiotic prescribing patterns for AOM • Contextual factors that may facilitate/impede watchful waiting • Contextual factors that may facilitate/impede implementation of intervention components	• Key informant interviews (clinicians and administrators) • Process mapping interviews (clinicians and administrators) • Electronic health record (EHR) data	• Adapt implementation plan to meet local needs and address barriers • Feedback baseline data to clinicians
Patient perspective	• Receptiveness to use of watchful waiting for AOM • Contextual factors that may facilitate/impede the acceptance of watchful waiting • Parent preferences for SDM process	• Key informant focus groups (parents)	• Adapt SDM process and education for clinicians to meet parent needs (Hybrid only) • Adapt communication education for clinicians • Adapt patient education materials
External environment	• HEDIS^a^ measures for antibiotic prescribing for acute respiratory tract infections • Cosmopolitanism	• Key informant interviews (clinicians and administrators) • EHR data to compute HEDIS measure	• Incorporate into clinician education and use for administrative buy-in if needed
Implementation and sustainability infrastructure	• Current resources and resource utilization for AOM management (e.g., EHR-tools, education materials) and stewardship • Baseline workflow for AOM • Perceived facilitators/barriers to sustainability	• Key informant interviews (clinicians and administrators) • Process mapping interviews (clinicians and administrators)	• Adapt sustainability plan and EHR workflow • Develop workflow for SDM (Hybrid only)
Organizational characteristics	• Existing collaborations and information flow between clinics • Administrative support • Shared goals	• Key informant interviews (clinicians and administrators)	• Modify communication plan and add administrative support if needed
Patient characteristics	• Demographics • Urban, suburban, rural designation	• EHR data collection • Clinic zip codes	• Use data for covariate-constrained cluster randomization

^a^ *HEDIS* Healthcare effectiveness data and information set

Contextual factors will be assessed at the child/parent, clinician, clinic, and health systems-levels using key informant semi-structured interviews and focus groups, process mapping, and electronic health record (EHR) data. Semi-structured key informant interviews will be conducted with a convenience sample of clinic administrators and clinicians across participating clinics to understand 1) typical workflow of visits for children with AOM, 2) contextual factors that may facilitate or impede use of watchful waiting, 3) contextual factors that may facilitate or impede implementation of intervention components, and 4) organization culture. To understand parent perspectives of factors that may facilitate or impede the acceptance of watchful waiting, focus groups of a convenience sample of parents from each site will be conducted.

Finally, to better understand variability in care, assist with setting targets for improvement, and inform the randomization of clinics, baseline EHR data will be abstracted to evaluate pre-intervention antibiotic prescribing patterns and patient-level characteristics from potential participating clinics. Substantial efforts will be made to minimize missing data on predictors by abstracting data from multiple EHR locations. For missingness of key predictors, sensitivity analyses will be carried out using multiple imputation approaches [41].

### Intervention components

The components of the health systems-level intervention are based on the CDC Core Elements of Outpatient Antibiotic Stewardship and broadly include engagement of a clinician champion at each participating clinic, changes to electronic health record to facilitate use of delayed antibiotic prescriptions, quarterly feedback reports detailing clinicians’ use of watchful waiting with comparison to their peers, and virtual learning sessions for clinicians [32]. The hybrid intervention will include the same elements as the health systems-level intervention with the addition of a shared decision-making aid and education sessions for clinicians regarding use of the aid. The interventions will be adapted based on the formative evaluation. A national stakeholder group comprised of representatives from the American Academy of Pediatrics (AAP), Centers for Disease Control and Prevention (CDC), and Pew Charitable Trusts provided input into the development of this work and will be convened at regular intervals over the study period to ensure that the interventions are low-cost, feasible to implement, generalizable, and sustainable.

### Clinician champions

Each participating clinic will be assigned a clinician champion to serve as the liaison for the study. The clinician champions will promote and advocate use of the assigned intervention tools as well as provide support and education for their peer clinicians throughout the intervention period.

### EHR tools

The participating clinics utilize three distinct EHRs – Epic, Cerner, and Athena. Together, these three EHRs account for over 75% of the EHR market in the United States. EHR changes will include updates to prescription fields to enable clinicians to more easily order a delayed antibiotic prescription as part of a watchful waiting approach to treatment. Patient education materials with watchful waiting instructions will be added to the EHR in languages appropriate for the patient populations (English, Spanish) at a fifth-eighth grade or below literacy level so that clinicians can include them in the after-visit summary. The information contained in the English and Spanish versions of the materials will be identical. Clinics will also have direct access to printable materials to accommodate their preferred method of dissemination.

### Audit and feedback reports for clinicians

Quarterly feedback reports detailing participating clinicians’ use of watchful waiting individually and compared with their peers will be created and shared using the Outpatient Automated Stewardship Information System (OASIS^©^). OASIS^©^ is a free, open-source method that uses statistical software to automate audit and feedback report creation and distribution and track clinician review of reports. The reports will show each clinician’s use of watchful waiting (observation or delayed antibiotic prescription vs immediate prescription) for AOM in children ≥ 6 months old in comparison to the same metric for peers in their clinic.

### Clinician education

Clinician education will include two optional one-hour virtual learning sessions. Education will focus on the clinical course of pediatric AOM with and without antibiotics, risks associated with antibiotics, and communication using the Dialogue Around Respiratory Treatment (DART) curriculum [42]. Learning sessions will be recorded. Continuing medical education (CME) and/or Maintenance of Certification (MOC) credit will be provided to participating clinicians. Clinicians who practice in clinics randomized to the hybrid health systems-level plus shared decision-making intervention will also receive access to an electronic or paper shared decision-making aid to use during AOM visits, along with education about use of the aid.

### Shared decision-making aid

The shared decision-making aid for AOM was developed and validated through observation of clinical care encounters, surveys of parents whose child recently had an ear infection, and interviews with parents [43]. The tool has been demonstrated to be patient-centered and have high internal and external validity. To further refine this tool, use of the shared decision-making aid will be recorded for observation by the research team during encounters with ten children with AOM. The findings will inform the adaptation of the aid along with input from a stakeholder advisory committee of parents, clinicians, and administrators to ensure the aid meets the needs of diverse patients and is easy to use for both parents and clinicians. The aid will be adapted for use on a webpage (current form) and on paper. The process of aid adaptation will align with the International Patient Decision Aid Standards [44, 45].

### Evaluation with RE-AIM including data analytics plan

The Reach Effectiveness Adoption Implementation Maintenance (RE-AIM) [34] framework will guide the evaluation of implementation outcomes (Table 4). To compare the effectiveness and implementation outcomes of the interventions, the Reach (how many and which type of patients were affected), Effectiveness (how many children took antibiotics and how many were managed with watchful waiting), Adoption (how many and which types of clinics participated), Implementation (fidelity, acceptability, appropriateness, time to implementation), and Maintenance (sustainability, feasibility of implementation in other sites) of interventions will be assessed. Contextual factors will be assessed at the child/parent, clinician, clinic, and health systems-levels. We will explore the differences in implementation effectiveness by patient population served, clinic type (pediatric, family medicine, urgent care), clinical setting (location, rurality, size), and organization. The fidelity, acceptability, and perceived appropriateness of the interventions among different clinician types, patient populations, and clinical settings will be compared.

**Table 4:** Outcome Measures using RE-AIM

	**Project Questions**	**Outcomes**	**How will data be obtained and at what intervals? (Comparable/similar measures or tools may be substituted). Time frames are approximate**
Reach	• How many patients can be reached by the interventions?	• Number of AOM episodes	• Electronic health record (EHR) data (baseline, quarterly during intervention, post-intervention)
Effectiveness	• Do the interventions increase parent satisfaction? • Do the interventions reduce antibiotic use for AOM?	• % very or extremely satisfied • % children take antibiotic (yes/no)	• Parent surveys (0, 10 days after diagnosis, throughout intervention) • Parent focus groups (pre- and post-intervention)
Adoption	• How many clinics will adopt the interventions? • Do the interventions require adaptations to meet local needs?	• Number/proportion of eligible clinics that agree to participate • Characteristics of clinics that participate vs. clinics that opt not to participate • Number/proportion of enrolled clinics that implement all program components	• AC, DHHA, and IH health system data (pre-intervention, post-intervention) • Clinician and administrator surveys (post-intervention) • Key informant qualitative interviews (pre intervention)
Implementation	• How many clinicians participate in intervention components? • Are the interventions acceptable to clinicians? • How effectively is SDM^a^ used? • How many children are managed with watchful waiting v immediate prescription? • How many parents fill antibiotic prescriptions (even if not taken by child)?	• Implementation of intervention components and time to implementation • Use of program components (fidelity) - Education sessions - Feedback reports - EHR tools - Clinician champion - Meet CME program requirements • Clinician perceptions of intervention component utility, acceptability, and appropriateness • Parent perception of SDM and appropriateness • % managed by immediate antibiotic • % parents fill antibiotic prescription	• Stages of Implementation Completeness [47] (SIC) tracking (quarterly through the study) • Attendance at education sessions, meeting CME Requirements, proportion of AOM episodes where EHR tools were used, proportion of feedback reports read by clinicians- tracked using read receipts [48] (quarterly during intervention and post-intervention) • Clinician and administrator surveys using the acceptability of intervention measure (AIM) and intervention appropriate measure (IAM) (post intervention [34] • Focus groups of parents (pre- and post-intervention)
Maintenance	• What is the likelihood of sustainability of the interventions? • Do clinicians and administrators think it would be feasible to implement the interventions in other settings? • Could the interventions be implemented in other settings?	• Clinical Sustainability Assessment Tool (CSAT) Sustainability Score [49] • Feasibility of intervention (FIM) measure [50]	• Clinician and administrator surveys using CSAT and feasibility of intervention measure (FIM) [50] (post-intervention)

^a^ *SDM *Shared decision-making

### Stakeholder engagement

We will seek input from key stakeholders to guide this research, increase the likelihood of success of the interventions, and disseminate results of the trial. A stakeholder advisory council comprised of parents, clinicians, and clinic administrators with representation from AC, DHHA, and IH will be assembled. The council will meet quarterly during the first year of the study, and twice per year over the remaining study period. The initial focus of the council will be understanding participants’ experiences and perceptions of watchful waiting, the creation of parent-facing education materials that are culturally appropriate and acceptable, and discussion of potential adaptations to the interventions to increase likelihood of success. Subsequent sessions will focus on addressing obstacles in study implementation, interpretation of results, and dissemination of findings. A national stakeholder group comprised of AAP, CDC, and Pew Charitable Trusts has provided input into the study design and will assist with interpretation and dissemination of results, dissemination of resources and tools developed through this work, and incorporation of findings into recommendations and guidelines.

### Patient eligibility, enrollment, and data collection

Children aged 6 months-17 years old treated for uncomplicated AOM at a participating clinic during the study period will be prospectively identified. Trained research staff will contact the parents of potentially eligible children via telephone, electronic mail, or the EHR and screen for eligibility. Children who received any antibiotic within 30 days prior to the visit, have a history of tympanostomy tubes, have current tympanic membrane perforation, or have a concomitant diagnosis that may warrant an antibiotic (e.g., Group A streptococcal pharyngitis, pneumonia) will be excluded. For children meeting all eligibility criteria, informed consent will be obtained from the parents (with assent from the child when appropriate) by trained research personnel. An example of the informed consent form can be found in the Supplement. Upon enrollment, the parents will complete a survey where the following data will be collected: demographic characteristics of the child (age, sex, race, ethnicity, language preference, insurance type), vaccination status, and treatment strategy used at the visit (observation, delayed antibiotic prescription, or immediate prescription). Ten days after enrollment, the parents will complete a second survey assessing whether any antibiotics that were prescribed were ultimately filled and taken, clinical encounters occurring after the index visit, new antibiotics prescribed within 30 days after the index visit, and their satisfaction with their child’s care.

To prevent potential imbalances in the number of children enrolled from a given clinic, there will be maximum number of children that can be enrolled from each clinic each month. Furthermore, there will be enrollment targets for race/ethnicity, preferred language, and insurance status. Participant recruitment has not yet begun (Table 5).

**Table 5:**
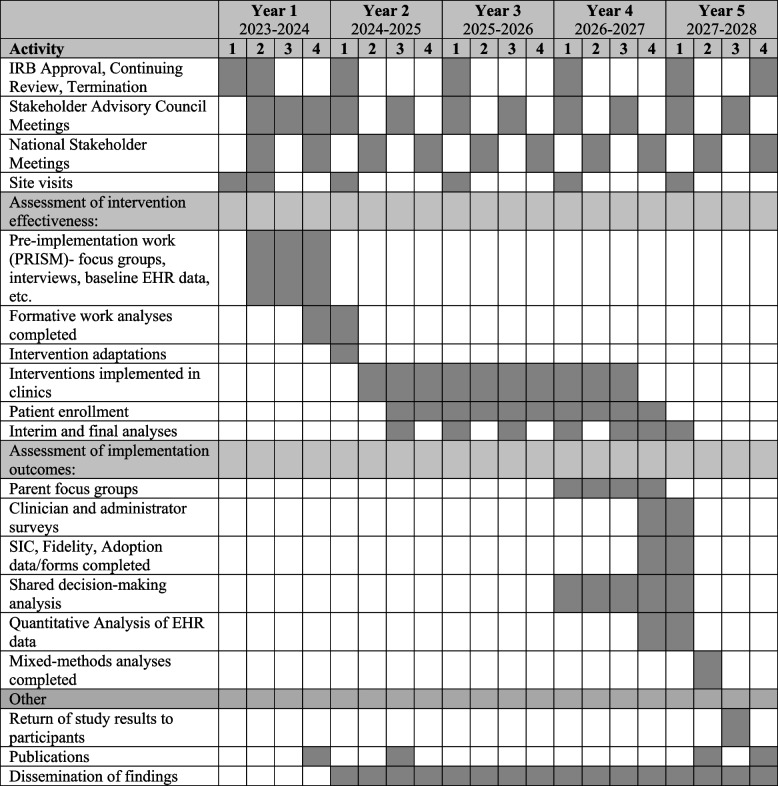
Detailed study timeline

To evaluate changes in antibiotic prescribing on a broader scale at the participating clinics, a secondary EHR analysis will be performed to determine the proportion of all children with an ICD-10 diagnosis of AOM meeting pre-specified eligibility criteria prescribed an immediate antibiotic (versus managed with observation or a delayed antibiotic prescription).

### Statistical analysis

The co-primary outcomes – whether the antibiotic was filled and taken and whether the parent was ‘very’ or ‘extremely satisfied’ with their child’s care – will be analyzed with a logistic mixed effects regression model. This model accounts for the hierarchical structure of the data with patients nested within clinics. The primary analysis will include covariates for the intervention group, the random effect for clinic, and pre-specified confounding factors including age, race, gender, ethnicity, language preference, and insurance type. Any variables that appear unbalanced between the study arms at *p* < 0.2 will also be included as predictors. Since it is possible that clinicians (within clinics) are another source of variation, an additional random effect for clinician within practices will be included if there is evidence of sufficient variability at this level. Multiple comparisons in testing the co-primary outcomes will be accounted for with an alpha = 0.025 to assess statistical significance. All other hypothesis tests will be two-sided with a significance level of 0.05.

Secondary outcomes (Table 1) will be analyzed using linear or logistic mixed effects regression, as appropriate. Similar random effects will be included accounting for the nested structure of the data. Secondary analysis results will be expressed as mean differences and regression coefficients or differences in proportion and odds ratios, as appropriate, with 95% confidence intervals (CIs).

Exploratory analyses will be conducted to test for potential effect modification by select patient characteristics. The effects of the health systems-level and hybrid interventions on antibiotic prescribing and parent satisfaction may differ based on race, ethnicity, insurance type, and clinical setting. Logistic mixed effects regression will be used including a main effect for the moderator and an interaction between the moderator and intervention variable. Due to the exploratory nature of these analyses, p-values will not be adjusted for multiple comparisons, however all subgroup analyses will be reported.

### Sample size and power

In the health systems-level intervention group, it is estimated the baseline proportion of cases where an antibiotic is taken will be 12% [19, 23, 24, 30] and 76% of parents will report being very or extremely satisfied [29]. Given the cluster design and co-primary outcomes, the sample size calculation used an intraclass correlation coefficient (ICC) of 0.02 to 0.05 for within-clinic correlation and a two-sided test with alpha = 0.025 to account for multiple comparisons. A total sample size of 1,566 children will be required to provide 80% power to detect an absolute difference in antibiotic courses taken of between 8% (ICC = 0.02) and 11% (ICC = 0.05) and an absolute difference in the proportion of parents satisfied between 9% (ICC = 0.02) and 12% (ICC = 0.05) when comparing the two intervention groups. Based on preliminary data from the 33 participating clinics, it is estimated there will be 27,997 eligible children across the sites over 3 years. With a projected 16% enrollment rate and 7% attrition rate [46], up to 4,100 children could successfully complete the study, well-exceeding the enrollment target of 1,566 children.

## Discussion

It is essential to develop interventions to optimize the management of children with AOM to reduce unnecessary antibiotic exposure, along with its inherent risks, and increase parent satisfaction with their child’s treatment. A watchful waiting management strategy for AOM offers a key opportunity to reduce antibiotic use for children with AOM; however, to date there have been no studies comparing the effectiveness of interventions to promote use of watchful waiting and parents’ perceptions and satisfaction with this approach. This study aims to fill the gap by evaluating the effectiveness and implementation outcomes of two pragmatic, patient-centered interventions – a health systems-level intervention with and without shared decision-making – that aim to reduce unnecessary antibiotic use for children with AOM while increasing parent satisfaction with their child’s care.

The strengths of this study include the cluster-randomized design, the mixed methods approach to guide implementation and evaluate outcomes and sustainability, and the diversity of the participating clinic systems. Additionally, components of the interventions are pragmatic and utilize tools that are freely available and feasible to implement in resource-limited settings. Another strength of the study is the use of surveys to accurately ascertain whether a prescribed antibiotic was actually filled and taken and to assess parent satisfaction with care. Given the pragmatic nature of the study, limitations will include the inability to assess whether included children who are diagnosed with AOM in clinical practice meet strict diagnostic criteria for AOM, the inability to assess the long-term sustainability of the intervention on prescribing practices, and the inability to determine the effects of changes in prescribing on antibiotic resistance. Nevertheless, this study will represent a significant contribution to the pediatric AOM literature where large, randomized, comparative effectiveness studies are lacking. It will provide a framework for other healthcare systems to reduce unnecessary antibiotic prescribing for AOM, engage parents and increase satisfaction with their child’s care, and inform national antibiotic stewardship policy development.

For further information about this study, please visit https://clinicaltrials.gov/study/NCT06034080.

### Supplementary Information

**Additional file 1. **Sample Consent Form.

## Data Availability

No datasets were generated or analysed during the current study.
