# Supplementary material for: Interventions to de-implement unnecessary antibiotic prescribing for ear infections (DISAPEAR Trial): protocol for a cluster-randomized trial
Source: BMC Infect Dis. 2024 Jan 24;24:126. doi: 10.1186/s12879-023-08960-z (PMC10807124; doi:10.1186/s12879-023-08960-z)
Supplement: Supplementary file 1 — Additional file 1. Sample Consent Form. [file 12879_2023_8960_MOESM1_ESM.docx]

**Supplement Section 1.** Sample Consent Form

This sample consent form will be tailored for use in different study components.

**Study Title:** DISAPEAR: Interventions to de-implement unnecessary antibiotic prescribing for children with ear infections

**Principal Investigator:**

**COMIRB No:**

**Version Date:**

You are being asked to be in this research study because you are ______. This study is taking place in clinics at AllianceChicago, Intermountain Healthcare, Mayo Clinic, and Denver Health and Hospital Authority. Denver Health and Hospital Authority is the lead study site and will lead the ____ and interpretation of the information you share with us.

If you join the study, you will be asked to participate in a _______.

The _____ is designed to help us learn more about ______.

There is possible discomfort associated with _____. There is a risk that ______. In an effort to protect your privacy and confidentiality, ______.

There may be risks the researchers have not thought of.

This study is not designed to benefit you directly, rather to improve care for children with ear infections. You will be paid ___ if you choose to participate.

This research is being paid for by Patient Centered Outcomes Research Institute (PCORI).

You have a choice about being in this study. You do not have to be in this study if you do not want to be.

The data we collect will be used for this study but may also be important for future research. Your data may be used for future research or sent to other researchers for future study without additional consent if information that identifies you is removed from the data.

If you have questions, you can call ______. You can call to ask questions at any time.

You may have questions about your rights as someone in this study. If you have questions, you can call the responsible Institutional Review Board at _____.

By participating in this ____ you are agreeing to participate in this research study.
